# Regional Anaesthesia, Contemporary Techniques, and Associated Advancements: A Narrative Review

**DOI:** 10.7759/cureus.65477

**Published:** 2024-07-26

**Authors:** Rutuja Gohad, Sudha Jain

**Affiliations:** 1 Anaesthesiology, Jawaharlal Nehru Medical College, Datta Meghe Institute of Higher Education and Research, Wardha, IND

**Keywords:** recent advancements, obstetric anaesthesia, spinal anaesthesia, ophthalmology, cancer, regional anaesthesiology, anaesthesia

## Abstract

In particular, the application of regional anaesthesia techniques in existing medicine can be characterized as experiencing regular changes in recent decades. It is useful for obtaining accurate and efficient pain management solutions, from the basic spinal and epidural blocks to the novel ultrasound nerve blocks and constant catheter procedures. These advancements do enhance not only the value of the perioperative period but also the patient's rated optimization as enhancing satisfaction, better precision, and the safety of nerve block placement. The use of ultrasound technology makes it even easier to determine the proper positioning of the needle and to monitor nerve block placement. Moreover, the duration and efficiency of regional anaesthesia are being enhanced by state-of-the-art approaches, which come in the form of liposomal bupivacaine, and better recovery plans and protocols, which shorten recovery time and decrease the number of hospital days. As these methods develop further, more improvements in the safety, efficacy, and applicability of regional anaesthesia in contemporary medicine are anticipated through continued research and innovation.

## Introduction and background

Both medical education and regional anaesthesia have completely changed during the last 20 years. From an apprenticeship paradigm, specialized training in regional anaesthesia has evolved to become a mandatory component of official graduate medical education programmes. The administration of local anaesthetic medications in specific areas of the body temporarily interrupts nerve conduction and pain perception without inducing unconsciousness, which is known as regional anaesthesia [1]. Methods of regional anaesthesia are widely used for treatments or procedures that are advantageous to the patient as well as the healthcare system as a whole. Therefore, healthcare providers must possess the essential skills to evaluate the success of perioperative medical interventions, including regional anaesthesia, within a comprehensive framework for healthcare improvement [2].

In 1987, Yeager and colleagues demonstrated a significant decrease in mortality among high-risk surgical patients using epidural analgesia. In 2000, Rodgers and colleagues conducted a comprehensive meta-analysis that indicated a reduction in postoperative mortality and morbidity associated with neuraxial anaesthesia (neuraxial anaesthesia is the placing of local anaesthetics in or near the central nervous system), prompting recommendations for its broader adoption in clinical practice [3]. The advancement and enhancement of regional anaesthetic methods for diverse surgical procedures, particularly in obstetric, ophthalmic, and orthopaedic surgeries, as well as the ongoing improvement of continuous regional analgesia, persist. In the training for regional anaesthesia within the field of anaesthesiology and intensive care medicine, emphasis on mastering essential techniques such as spinal block, epidural block, axillary brachial plexus block, femoral nerve block, and intravenous regional anaesthesia is typically considered comprehensive [4]. Generally, performing more peripheral blocks tends to reduce the occurrence of complications and adverse effects [5]. Peripheral regional anaesthesia is a crucial aspect of contemporary perioperative care. Peripheral regional anaesthesia can be effectively performed with simple techniques, such as fascia iliaca compartment blocks, requiring minimal technological resources. Among other methods, ultrasound imaging has gained broad clinical acceptance in this field to guide the needle movement towards the nerves, minimizing the risk of needle contact with critical structures and reducing potential complications [6]. Numerous publications extensively cover different facets of peripheral regional anaesthesia techniques as applied in routine clinical settings [7].

The field of anaesthesiology stands to gain significantly from advancements in artificial intelligence (AI), as it spans various aspects of clinical care, such as perioperative and intensive care management, pain relief, and innovation in drug administration and development [8]. Simulation-based assessments in anaesthesiology utilize computer-assisted case management simulations, mannequins, and task trainers for both formative and summative purposes. While the initial focus was on part-task trainers such as airway management tools and full-body electromechanical mannequins, extensive research has significantly progressed in the field of performance assessment in anaesthesiology [9].

In anaesthesiology, AI technologies can profoundly influence every stage of perioperative care, from initial surgical planning and pre-anaesthetic evaluation to intraoperative monitoring and postoperative care, ultimately enhancing care quality and improving patient outcomes [10]. Jane McGonigal's insights suggest that video games can effectively showcase personal strengths, help users achieve goals, enhance motivation, and stimulate creativity. Integrating gamification into anaesthesia, critical care, and pain medicine presents a promising avenue. Its dynamic and interactive nature, coupled with its significant effectiveness, can enrich learning experiences, improve non-technical and technical skills, and contribute positively to universal advancements in these fields [11].

Since its introduction in 1989, ultrasound-guided imaging has become the primary method for precisely directing the blockade of peripheral nerves in regional anaesthesia [12]. Mastering ultrasound-guided regional anaesthesia early in training poses significant challenges. Machine learning systems can potentially offer supportive tools to assist inexperienced operators in acquiring the necessary skills for ultrasound-guided regional anaesthesia [13]. The field of anaesthesiology stands poised to harness potential benefits from advancements in AI, impacting various aspects of patient care such as intensive care and perioperative management, pain therapy, and advancements in the administration of drugs and discovery [8]. In 1950, Bickford initiated one of the first efforts in AI history by utilizing electroencephalogram (EEG) signals for monitoring and maintaining the depth of anaesthesia. In 2008, the McSleepy AI machine was developed, featuring an automated anaesthesia system trained using machine learning, specifically adaptive neural networks, to execute tasks through input-output mapping [14]. In a recent study by Shamberg et al. (2022), continuous action deep reinforcement learning demonstrated superior performance compared to proportional integral derivative model control in administering anaesthesia drugs. Additionally, reinforcement learning generated anaesthesia control strategies that are easily interpretable [15].

The advancement of AI in medicine relies heavily on patient data, underscoring the crucial need for robust ethical practices and regulations that prioritize patient privacy and well-being. As new AI-driven interventions leveraging medical records emerge, it is essential to uphold ethical and humanistic principles, especially when collaborating with external researchers or companies [16].

## Review

Table 1 shows a description of the articles reviewed in the study.

**Table 1:** Summary of the studies included in the review

Author	Year	Type	Conclusion
Ramlogan et al. [1]	2020	Review article	The review investigated educational strategies employed in dedicated regional anaesthesia rotations for trainees, alongside revolutions in teaching new ways for advanced clinical fellows or consultants. While each modality contributes to learning benefits, it is evident that no single approach is sufficient alone to encompass the diverse skills needed for performing and managing regional anaesthesia.
Johnston and Turbitt [2]	2020	Review article	The study found that the success of a regional anaesthesia technique depends on multiple factors, including patient characteristics, population demographics, healthcare system considerations, and training advantages. Identifying a select set of high-value nerve blocks could promote a more standardized, widespread, and effective approach to delivering regional anaesthesia on a global scale.
Kettner et al. [3]	2011	Review article	According to the study, existing outcome studies and meta-analyses do not account for individual skills and their direct correlation with success rates. The author suggests that failed blocks likely represent the most significant factor contributing to negative outcomes. Enhancing individual practitioner skills and employing regional anaesthesia judiciously and appropriately can likely lead to improved outcomes by reducing failure rates.
Garg [17]	2017	Editorial	In summary, maintaining perioperative homeostasis through balanced anaesthesia, which includes regional blocks, may have protective effects against cancer recurrence and other beneficial outcomes. Continuing randomized controlled trials aimed at assessing the influence of regional anaesthesia on oncological outcomes will offer conclusive guidance in the future.
Bithal and Rath [18]	2021	Editorial	The discussion underscores the underutilization of regional anaesthesia as a valuable tool in the neuroanaesthesiologist's toolkit. Utilizing regional anaesthesia in cooperative patients or those with multiple systemic comorbidities could enhance patient outcomes while conserving hospital resources.
Cascella et al. [11]	2023	Review article	The study concluded that integrating gamification strategies in these intricate clinical domains shows promise for substantial advancements, encompassing both simulated and real-world scenarios. This dynamic approach has the potential to significantly improve knowledge retention, skill acquisition, and overall performance, enhancing both technical and non-technical competencies.
Jaichandran [19]	2013	Review article	The study concluded that the selection of technique should be tailored to meet the unique requirements of the patient, the type and complexity of the eye surgery, and the preferences and expertise of the anaesthesiologist and surgeon. However, a comprehensive understanding of orbital anatomy and proper training are essential for safely administering orbital regional anaesthesia.
Boulet and Murray [9]	2010	Review article	The study highlights that through the integration of simulation-based training and robust assessments, the specialty has acknowledged the importance of advancing professional skill development. This commitment reflects a sustained dedication to enhancing patient care, addressing challenges, and ensuring psychometrically sound evaluations.

Regional anaesthesia in neuro-anaesthesia practice

Neurosurgical anaesthesiology, a relatively recent subspecialty within anaesthesiology, specializes in managing anaesthesia for patients undergoing cranial and spinal neurosurgical procedures. Neurophysiological monitoring is commonly employed during spinal procedures to guide the selection and administration of general anaesthetic drugs. Postoperative pain following spinal surgeries, particularly in the initial days, can be intense [20]. Regional anaesthesia has been shown to potentially lower the incidence of persistent postoperative pain (PPP), a common and debilitating issue [21]. Effective pain management through regional anaesthesia techniques correlates positively with enhanced functional recovery, early mobilization, shorter hospital stays, and reduced risk of chronic pain development. Therefore, the judicious use of regional anaesthesia not only supports intraoperative management but also enhances overall postoperative outcomes for neurosurgical patients [20].

During neurosurgery, anaesthesiologists predominantly use opioids for intraoperative pain relief. However, opioids can potentially prolong recovery from anaesthesia, hindering prompt neurological assessment in the immediate postoperative period. Harvey Cushing and George Crile first described the combination of local anaesthetic infiltration of the scalp with general anaesthesia for craniotomies in the early 1900s. Advancements in modern neuro-anaesthesia, including enhanced neuromonitoring capabilities and safer anaesthetic agents, have limited the application of regional anaesthesia in neurosurgical patients. Scalp nerve block (SNB) serves as the primary regional anaesthesia technique for awake craniotomy and postoperative pain management. It involves blocking bilateral branches of the trigeminal nerves. SNB is effective and provides long-lasting postoperative pain relief in both adults and paediatric patients. Scalp block has been linked to enhanced progression-free survival in patients undergoing primary glioma resection. By minimizing intraoperative opioid use, scalp block supports prompt neurological assessment in the immediate postoperative period. Spine surgery, a substantial component of neurosurgical procedures, ranges from straightforward interventions (such as single or two-level discectomies) to more intricate procedures involving instrumentation. Neuraxial anaesthesia presents an appealing option for single or two-level discectomy procedures. Epidural anaesthesia can also serve as an alternative to spinal anaesthesia in uncomplicated lumbar spine surgeries. Neuraxial anaesthesia offers cost savings for both hospitals and patients, along with benefits such as decreased blood loss, reduced hemodynamic instability, decreased postoperative analgesic requirements, shorter hospital stays, lower incidence of urinary retention, reduced occurrence, and a lower incidence of thromboembolism [18].

Neurosurgical patients require specialized airway management. Airway management in neuro-anesthesia generally follows principles similar to those of other anaesthesia fields. However, specific factors related to neurosurgical procedures and underlying neurological conditions can pose challenges in maintaining ventilation and airway patency. Immediate indications for neurosurgical intervention include persistent apnea, airway obstruction, or ventilation difficulty. Urgent situations arise when Glasgow Coma Scale (GCS) score is 8 or lower or there is anticipated occlusion due to haematoma, oedema, or laryngotracheal injury. Relative indications may involve the need to manage intracranial pressure through pCO_2_ control [22].

Regional anaesthesia in cases of labour, operative vaginal delivery, and caesarean delivery

Spinal anaesthesia is preferred for caesarean sections, particularly in elective cases, as it mitigates common perils linked with general anaesthesia, including aspiration, difficult intubation, and the potential adverse impact of general anaesthetics on the foetus. Optimizing treatment strategies for achieving haemodynamic stability throughout spinal anaesthesia for caesarean section remains a key challenge in obstetric anaesthesiology [23]. The use of neuraxial analgesia is still regarded as the standard for labour and delivery across the world. As many as 71% of the US use some form of neuraxial analgesia. Epidural and combined spinal-epidural are two very popular ways of administering anaesthesia for labour [24]. Combined spinal epidurals have been used to some extent in recent years because the medicine given during intraoperative anaesthesia can also be continued in the postoperative period by placing the epidural catheter [25].

Pain is prevalent during labour and delivery, and it remains a significant concern for women after caesarean delivery. Addressing postpartum pain is crucial for promoting recovery across all modes of delivery. Anaesthetists' increased knowledge and access to ultrasound for regional blocks are increasing the usage of obstetric regional anaesthesia. Analgesic nerve blocks in vaginal and operative vaginal delivery rely more on obstetricians who commonly perform paracervical and pudendal nerve blocks, unlike anaesthetists. Paracervical nerve blockade involves the infiltration of local anaesthetic solution into the submucosa of the cervical fornix, where it blocks the paracervical ganglion and thus controls pain from the cervix as well as the uterus. Compared to placebo or opioid analgesia, paracervical blockade consistently results in higher satisfaction with analgesia and lower pain scores. Paracervical blockade is recognized for its superior efficacy in providing labour analgesia compared to placebo and opioids.

Moreover, during the second stage of labour and after the episiotomy, to relieve maternal pain, a local block of the pudendal nerve can be given. The pudendal nerve that arises from the S2 to S4 deals with innervations of structures in the perineal region, vulva, and the lower segment of the vagina. Administering a pudendal nerve block offers effective pain relief during the second stage of labour and vaginal delivery. However, it is not effective in eradicating pain linked to contractions occurring in the first stage of labour. This is due to the popularity of epidural services, which has seen a reduction in the use of techniques such as paracervical nerve blockade in labour and pudendal nerve blockade in operative vaginal deliveries. Among caesarean delivery patients, the transverse abdominis plane (TAP) blockade is the most extensively researched truncal nerve block. There are several approaches to the TAP compartment, and the techniques differ [26]. Utilizing ultrasound guidance enhances the precision of needle placement. When conducted by a surgeon with direct visualization of intra-abdominal structures during the procedure, the TAP block is notably quicker to perform and yields comparable analgesic outcomes compared to blocks administered by an anaesthetist [27].

Ophthalmic regional anaesthesia

For nearly a millennium, eye surgeries were conducted with minimal or absent use of anaesthesia. The year 1884 marked a significant advancement in ophthalmic anaesthesia with Carl Koller. The findings include the uses of cocaine hydrochloride in emerging ophthalmologic procedures such as topical application in eye surgery and further application of the same in retrobulbar injections by Herman Knapp as well as in the enucleation process. Nonetheless, it is important to recognize that topical anaesthesia has inherent limitations [19]. There has been a shift in the delivery of anaesthesia from general anaesthesia towards locoregional anaesthesia (LA) techniques for several reasons: greater organizational performance and its value objectives in efficiency, cost, and service, saving patients, and improving satisfaction [28]. The decision on which type of LA to use in ophthalmic surgery and where to apply it depends on the surgery undertaken, the duration of time required for the specific surgical intervention, and the patient’s profile. Topical anaesthesia is a rapid and straightforward technique. However, it is by far from injections with their possible dangers. Local anaesthetic subconjunctival injection entails the instillation of the injectable under the conjunctiva. A sub-tenon anaesthesia can be defined as the process of injecting local anaesthetic in the sub-tenon space, that is, the space happening between the tenon capsule and the sclera. In intended intraocular procedures, intracameral prescription of anaesthesia entails the instillation of a small amount of anaesthetic (0.1-0.5 mL) directly into the AC. Selective nerve blocks can be given through two mechanisms: conventional technique involving local injections directly into a specific area or provided through a regional nerve blockade [29].

Peribulbar and retrobulbar anaesthesia are the most common ways of making cataract surgery into a day surgical procedure [30]. In the modern retrobulbar block technique, a 23-gauge needle measuring 31 mm in length is inserted through the skin in the inferotemporal quadrant. The needle's bevel faces towards the globe and is positioned as laterally as possible, just above the junction of the inferior and lateral orbital walls. Starting from the outermost corner, this approach ensures ample distance from the globe, minimizing the risk of accidental injury to the inferior rectus muscle or its neurovascular bundle. Approximately 4-5 cc of local anaesthetic is then injected with the eye positioned in the primary gaze. This technique effectively paralyzes all extraocular muscles except the superior oblique. It achieves comprehensive globe anaesthesia by blocking both the nasociliary and long ciliary nerves. A 25 mm long, 23-gauge needle is placed as laterally as possible in the inferotemporal quadrant during the contemporary peribulbar block method. Using the eye in the main gaze, 4-5 cc of local anaesthetic is given after aspiration is used to confirm the absence of blood. This facilitates the efficient diffusion of the local anaesthetic solution through the orbital septum, which may also cause paralysis of the orbicularis muscle.

For the medial peribulbar block, a disposable half-inch needle with a gauge of 26 is used. The needle is inserted into the depression between the medial caruncle and the canthus, with its angle towards the medial orbital wall; 3-5 cc of a local anaesthetic solution is injected once it has been confirmed that no blood has been aspirated. This extraconal space is optimal for administering local anaesthesia, as it freely communicates with the intraconal space. Additionally, the injection into this area can lead to the eyelids filling with the anaesthetic solution, achieving excellent orbicularis muscle akinesia [19]. Since peribulbar anaesthesia has a prolonged latency, it is advantageous to perform the block at least 10 minutes prior to the commencement of surgery [31].

Regional anaesthesia in breast cancer

Because of the extended time for peribulbar anaesthesia onset, it is better to perform the block no later than 10 minutes before the start of surgery [32]. Onco-anaesthesia essentially encompasses the American Society of Anesthesiologists (ASA) guideline concerning the management of cancer patients for a timely resumption or initiation of the planned oncological treatment with a view to reducing cancer relapse and improving patients' survival rate [33]. In oncological surgery, regional anaesthesia has been recommended due to certain rationalizations for the mechanism of preventing cancer-related cell implantation and growth, which occur through several approaches [34]. Treatment for breast cancer often involves surgical procedures such as lumpectomy or mastectomy, which may include axillary lymph node dissection.

Regional anaesthesia offers efficient anaesthesia and pain relief during the perioperative period for breast surgeries. Local wound infiltration is also employed as a technique, as well as thoracic epidural anaesthesia, thoracic paravertebral block, thoracic spinal anaesthesia, and other techniques that have recently emerged, such as ultrasound-guided interfascial plane blocks such as pectoral nerve blocks (type 1 and 2) and serratus plane block (SPB). Sedation is thought to offer the capability to stop cancer cells from spreading by reducing the surgical stress response system, effectively managing pain, reducing opioid dependency to this method, and directly ruling out metastatic cell movement. Such anaesthetics are believed to be effective in preventing ever-recurrence of cancer because they cause a direct inhibition of cancer growth and possess indirect effects on cell division. Local anaesthesia helps maintain immune function by avoiding the use of analgesics and preventing surgical stress response. One benefit of this type of anaesthesia is that it can reduce the need for opioids and volatile anaesthetics. Another benefit of localized analgesia is a decreased incidence of postoperative nausea and vomiting (PONV) [17].

Paravertebral block has been demonstrated in retrospective investigations to be useful in reducing recurrence and chronic pain. Alternative nerve blocks for post-mastectomies include pec and erector spinae blocks [35]. Regional anaesthesia has been thought to benefit the immune system by reducing postoperative pain because uncontrolled pain has also been demonstrated in animal studies to decrease NK cells and encourage metastasis [32]. Preoperative regional blocks have shown efficacy in lowering chronic pain following breast cancer surgery, according to a meta-analysis and Cochrane review focusing on persistent pain after breast surgeries. With these benefits, regional anaesthesia methods for breast surgery may make them more practicable in an outpatient situation. In conclusion, in addition to other advantages, preserving perioperative stability with a balanced anaesthetic strategy that includes localized blocks may protect against cancer recurrence. Significant information for upcoming clinical recommendations will emerge from ongoing randomized controlled trials examining the effect of regional anaesthesia on oncological outcomes [17].

## Conclusions

It can be concluded that regional anaesthesia has evolved significantly with modern approaches, and the novelty they offer to healthcare practitioners and patients, as well as their numerous benefits over traditional methods, are undeniable. These stipulated innovations include extended peripheral nerve block catheters, the use of ultrasonic irradiation in nerve blocks, and the evolution of local anaesthetics to attain enhanced precision, security, and efficacy in regional anaesthesia. These innovations have been made possible by a more efficient management of pain, a reduction in the use of opioids, and an increased rate of patients' recovery.

According to expectations, regional anaesthesia will expand even more in the future due to science and technology proving that it is an essential part of patient care as well as modern-day medicine. Additionally, the versatility of regional anaesthesia has also expanded, as regional anaesthetics benefit from innovative local anaesthetics with a longer half-life and less toxicity. The UFC has become even better due to such techniques as nerve stimulation and the use of supplementary drugs when preparing for the procedure and when making pain management plans. They contribute to reducing hospital stays and the time it takes for patients to regain their strength and well-being, increasing the satisfaction of healthcare consumers.
